# Current insights into bacterial secondary infection following influenza A virus infection

**DOI:** 10.3389/fmicb.2026.1851115

**Published:** 2026-05-25

**Authors:** Jeong-Hoo Seo, Ye-Ji Seo, Hong-Yeoul Ryu, Minsang Shin, Chung-Young Lee

**Affiliations:** 1 Department of Microbiology, School of Medicine, Kyungpook National University, Daegu, Republic of Korea; 2 BK21 FOUR KNU Creative BioResearch Group, KNU G-LAMP Research Center, School of Life Sciences, College of Natural Sciences, KNU Institute of Basic Sciences, Kyungpook National University, Daegu, Republic of Korea; 3 Untreatable Infectious Disease Institute, Kyungpook National University, Daegu, Republic of Korea

**Keywords:** dysbiosis, immune dysregulation, influenza A virus, multidrug-resistant pathogens, secondary bacterial infection

## Abstract

Influenza A virus (IAV) continues to pose a substantial challenge to global health, not merely through primary viral pneumonia but largely due to lethal secondary bacterial complications. Pathogens such as *Streptococcus pneumoniae*, *Staphylococcus aureus*, and *Haemophilus influenzae* capitalize on the physiological “storm” induced by IAV, leading to significantly exacerbated morbidity. This review provides a comprehensive synthesis of the multifaceted mechanisms that dismantle host antibacterial defenses. Beyond the classical understanding of respiratory epithelial damage and the compensatory upregulation of bacterial adhesion receptors, we delve into the sophisticated dysregulation of innate immune signaling, specifically the collateral damage caused by interferon responses and impaired phagocytic function. Furthermore, we examine the complex roles of direct virus–bacterium synergism and the disruption of the respiratory microbiome (dysbiosis). By integrating these established paradigms, we extend the discussion to the rising clinical concern of nosocomial and multidrug-resistant (MDR) infections in critically ill patients. We conclude by identifying critical knowledge gaps and emphasizing the need for targeted strategies to mitigate the host vulnerabilities that permit opportunistic MDR colonization in the wake of viral insult.

## 1 Introduction

**Figure 1:**
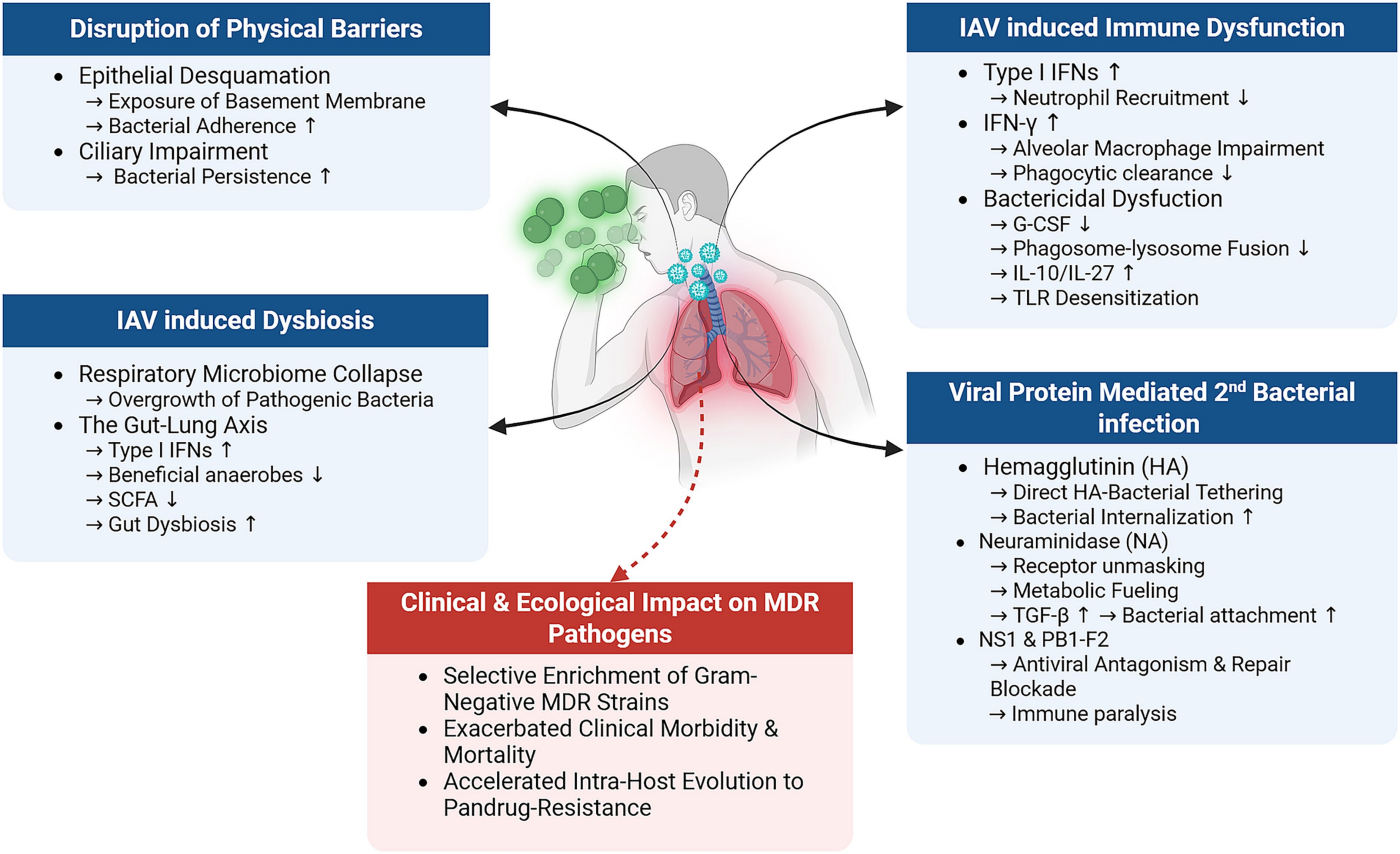
Multifaceted mechanisms of IAV-induced susceptibility to secondary bacterial and MDR infections. Influenza infection dismantles host defenses through primary, interconnected axes, ultimately facilitating colonization, invasion, and the potential for increased drug resistance in secondary pathogens. In the central illustration, green spheres represent secondary bacteria, while blue particles indicate IAV virions. This synergy is driven by several key mechanisms. The disruption of physical barriers occurs via epithelial desquamation and ciliary impairment. IAV-induced immune dysfunction is characterized by dysregulated cytokine responses and impaired phagocytic clearance. Additionally, IAV-induced dysbiosis emerges in both respiratory and gut-lung axes. Viral protein-mediated interactions, where HA and NA facilitate bacterial attachment and fueling, further exacerbate the infection. These processes collectively lead to the selective enrichment of MDR strains and increased morbidity (created with BioRender.com).

Influenza A viruses (IAV) are enveloped, single-stranded RNA viruses belonging to the *Orthomyxoviridae* family. Clinically, infection typically manifests as an acute respiratory illness characterized by fever, cough, myalgia, headache, and malaise. The severity of the clinical course is primarily dictated by the delicate balance between viral replication kinetics and the host’s inflammatory response. Rapid and extensive viral propagation within the lower respiratory tract can trigger a cytokine storm, a dysregulated surge of pro-inflammatory mediators (Yokota, 2003; Tisoncik et al., 2012). This hyper-inflammatory state inflicts substantial collateral damage on the lung parenchyma, leading to diffuse alveolar damage and disruption of the respiratory epithelium. Furthermore, the robust interferon (IFN) signaling required for viral clearance paradoxically induces a transient refractory state in the innate immune system, characterized by impaired recruitment and diminished phagocytic function of neutrophils and alveolar macrophages (Sun and Metzger, 2008; Shahangian et al., 2009). This multifaceted collapse of physical and immunological barriers creates a highly permissive environment for opportunistic bacterial pathogens (Sun and Metzger, 2008; Goulding et al., 2011; Ghoneim et al., 2013) (Figure 1).

Historically and clinically, the hallmark of severe influenza lies in its association with secondary bacterial infections (Morens et al., 2008). For instance, it is now widely recognized that the majority of deaths during the 1918 influenza pandemic were attributed not to primary viral insult, but to secondary bacterial pneumonia (Morens et al., 2008). This trend is still evident in modern seasonal and pandemic influenza in which bacterial coinfection remains a leading cause of hospitalization and mortality (Rice et al., 2012). Globally, influenza-related deaths reach approximately 650,000 annually, with influenza-associated complications accounting for 14.1% of adult hospitalizations as of 2021 (Lafond et al., 2021; Chen et al., 2026). Recent studies indicate that the 30-day mortality and ICU admission rates for influenza-bacterial coinfections reach 10.9 and 9.5%, respectively, which are significantly higher than those observed in viral monoinfections (Liu et al., 2021; Chen et al., 2026). Furthermore, a meta-analysis of IAV-infected patients revealed that approximately 11.2% were diagnosed with a concomitant bacterial infection, which increased the mortality of hospitalized patients 3.4-fold (Arranz-Herrero et al., 2023). Traditionally, the primary bacterial pathogens responsible for this synergy have been well-documented community-acquired agents, such as *Streptococcus pneumoniae*, *Staphylococcus aureus* (including MRSA), and *Haemophilus influenzae* (Iverson et al., 2011; Smith and Huber, 2018). According to recent data, *S. pneumoniae* (30.66%) and *S. aureus* (30.41%) remain the most prevalent causes, followed by *H. influenzae* (7.11%) (Arranz-Herrero et al., 2023). Notably, multidrug-resistant (MDR) pathogens including *Pseudomonas aeruginosa* (5.88%) and *Acinetobacter baumannii* (4.11%) are also frequently identified, representing a non-negligible portion of the clinical landscape. This presence of MDR strains highlights the increasing complexity of managing secondary bacterial complications in modern intensive care.

The shifting landscape of clinical medicine presents a new and substantial challenge. With the advancement of modern intensive care and the global escalation of antimicrobial resistance, the spectrum of post-influenza complications expands beyond classical pathogens to include nosocomial and MDR infections (Liu et al., 2018). Emerging evidence suggests that influenza-induced host vulnerabilities similarly facilitate the invasion of highly virulent opportunistic MDR pathogens, such as *Acinetobacter baumannii* (Liu et al., 2018). These infections pose a distinct therapeutic dilemma because of their limited treatment options and high mortality rates in critically ill patients (Jia et al., 2017). Therefore, this review aims to provide a comprehensive synthesis of the multifaceted mechanisms underlying viral–bacterial synergism. We explore both established pathways involving classical pathogens and the emerging threat of MDR-associated secondary pneumonia, establishing a theoretical framework for future clinical and translational research.

## 2 Mechanisms driving increased bacterial susceptibility following influenza

### 2.1 Remodeling of the respiratory surface: structural damage and hijacked adhesion

Influenza virus infection predisposes the host to secondary bacterial complications primarily through the structural disintegration of the respiratory epithelium, which markedly facilitates bacterial adherence (McCullers, 2006, 2014). Viral replication exerts a direct cytopathic effect on the respiratory epithelium by hijacking host protein synthesis and triggering programmed cell death pathways, including apoptosis and necrosis (Burri, 1975; Smith and Huber, 2018; Gaucherand et al., 2019). These events culminate in extensive epithelial desquamation (Morens et al., 2008). In severe cases, the epithelial lining is stripped down to the basal cell layer, thereby exposing the basement membrane and previously sequestered adhesion sites (Kash et al., 2011). This loss of epithelial integrity renders the extracellular matrix components, such as laminin, type IV collagen, and fibrinogen, accessible to opportunistic pathogens (Jia et al., 2017). Crucially, the viral neuraminidase (NA) cleaves terminal sialic acid residues from host glycans to unmask cryptic receptors (McCullers and Bartmess, 2003; Peltola et al., 2005). This process promotes the stable attachment and invasive dissemination of secondary pathogens, most notably *S. pneumoniae* and *S. aureus* (McCullers and Bartmess, 2003; Peltola et al., 2005; Li et al., 2015).

Influenza A virus (IAV) further promotes bacterial invasion by co-opting host cellular machinery (Bai et al., 2021; Sumitomo et al., 2021; Okahashi et al., 2022). A prominent example is the host chaperone protein GP96, which undergoes IAV-induced ectopic translocation from the endoplasmic reticulum (ER) to the plasma membrane (Sumitomo et al., 2021). In this atypical surface location, GP96 acts as a molecular scaffold that physically bridges host αV integrins with bacterial AliA/AliB proteins (Sumitomo et al., 2021). This interaction provides a robust molecular foothold for *S. pneumoniae* colonization (Sumitomo et al., 2021). Furthermore, it has been demonstrated that pharmacological inhibition of GP96 with PU-WS13 effectively abrogates pneumococcal adherence and enhances *in vivo* bacterial clearance, highlighting its potential as a therapeutic target (Patel et al., 2013; Sumitomo et al., 2021).

The mucociliary escalator constitutes a critical mechanical defense of the respiratory tract by trapping inhaled microbes within mucus and expelling them through coordinated ciliary rhythmic activity (Wanner et al., 1996; Pittet et al., 2010). Influenza infection inflicts profound structural and functional ciliary impairment, primarily via the direct lysis of ciliated cells (Pittet et al., 2010). Furthermore, IAV infection significantly compromises mucociliary clearance (MCC) by disrupting the planar cell polarity of the remaining ciliated epithelium (Pittet et al., 2010). This molecular dysregulation leads to reduced ciliary beat frequency and asynchronous, disorganized motion, resulting in a marked decrease in tracheal mucociliary velocity (Nugent and Pesanti, 1983; Pittet et al., 2010). Such functional failure substantially extends the residence time of bacterial pathogens within the airways, facilitating the colonization of species such as *S. pneumoniae* (Pittet et al., 2010). Pittet et al. (2010) demonstrated that the cessation of mucociliary movement correlates with a 220-fold increase in bacterial burden by day 6 post-infection. This impaired clearance not only drives local bacterial accumulation but also facilitates the aspiration of pathogens from the upper respiratory tract into the lower airways, ultimately precipitating secondary bacterial pneumonia (Siegel et al., 2014). When necrotic debris, hypersecreted mucus, and inflammatory exudates consolidate, they obstruct small airways (Burri, 1975; Lin and Zhang, 2019). This creates protected, nutrient-rich niches that enhance bacterial persistence and facilitate deeper migration into the lung parenchyma (Siegel et al., 2014).

### 2.2 IAV-induced immune dysfunction

**Figure 2:**
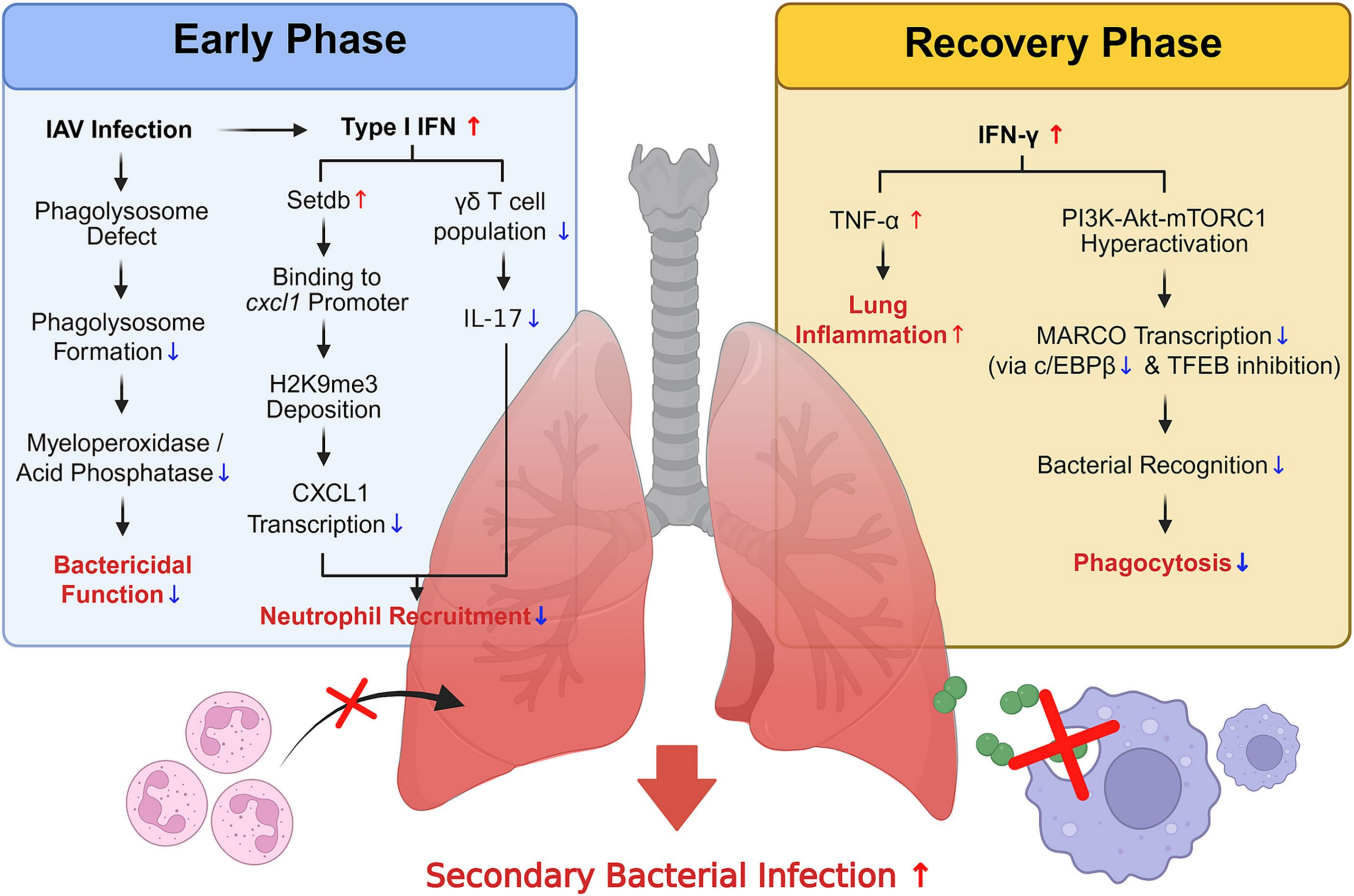
Impairment of innate immune responses by IAV infection predisposing to secondary bacterial infection. During the early phase of IAV infection, elevated type I IFNs (IFN-α/β) suppress the expression of CXCL1 and IL-17, which leads to the inhibition of neutrophil recruitment and impaired bactericidal activity. In the recovery phase, upregulated IFN-γ signaling results in the hyperactivation of the PI3K-Akt-mTORC1 pathway, subsequently suppressing MARCO transcription via inhibition of c/EBPβ and TFEB. This cascade reduces the phagocytic capacity of macrophages. Concurrently, altered TNF-α dynamics contribute to persistent or exacerbated lung inflammation, collectively increasing susceptibility to secondary bacterial infections (created with BiorRender.com).

The initial response to IAV is dominated by the rapid induction of Type I Interferons (IFN-α/β), which limit viral replication but paradoxically undermine the host’s ability to mount an effective innate defense against secondary bacterial challenges (Shahangian et al., 2009; Lee et al., 2015) (Figure 2). IAV-induced Type I IFN signaling potently upregulates the lysine methyltransferase Setdb2 in myeloid cells, including macrophages (Schliehe et al., 2015). Setdb2 occupies the promoter of the neutrophil chemoattractant *Cxcl1* and catalyzes the trimethylation of histone H3 lysine 9 (H3K9me3) (Schliehe et al., 2015). This repressive epigenetic mark leads to transcriptional silencing of CXCL1, directly resulting in decreased neutrophil infiltration during subsequent bacterial superinfection (Schliehe et al., 2015). Additionally, the induction of type I IFNs negatively regulate γδ T cells population, which are the primary early source of IL-17 in the lungs (Li et al., 2012). The resulting deficiency in IL-17 prevents the efficient recruitment of neutrophils to the infection site (Kudva et al., 2011; Li et al., 2012). In addition to recruitment defects, the innate effector functions of the resident immune cells are directly impaired. IAV infection causes defective phagolysosome formation (Jakab et al., 1980; Abramson et al., 1982; Metzger and Sun, 2013). This ablation of normal intracellular lysosome-phagosome fusion leads to a significant decrease in the intraphagosomal release of myeloperoxidase (MPO) and acid phosphatase, ultimately depressing the bactericidal functions of polymorphonuclear leukocytes (Abramson et al., 1982).

During the recovery phase of influenza infection, typically 7–8 days post-infection, infiltrating T cells produce large amounts of IFN-γ (Sun and Metzger, 2008). While T cells are involved in the resolution of the viral infection, the ensuing IFN-γ milieu drives a profound functional paralysis and depletion of alveolar macrophages (AMs) (Sun and Metzger, 2008; Ghoneim et al., 2013; Verma et al., 2020) (Figure 2). High levels of IFN-γ induce the hyperactivation of the PI3K-Akt-mTORC1 pathway within AMs, which suppresses the expression of MARCO (Macrophage Receptor with Collagenous structure), a class A scavenger receptor essential for the recognition and nonopsonized phagocytosis of extracellular bacteria such as *S. pneumoniae* (Sun and Metzger, 2008; Wang et al., 2015; Wu et al., 2017). Mechanistically, mTORC1 hyperactivation blocks MARCO transcription through two parallel axes (Wang et al., 2015; Wu et al., 2017). mTORC1 activation decreases the expression of the transcription factor c/EBPβ, which directly binds to the MARCO gene promoter to induce its expression (Wang et al., 2015). Furthermore, mTORC1 overactivity leads to the inhibition of downstream Akt signaling (Wu et al., 2017). This suppression results in the phosphorylation and cytoplasmic sequestration of the transcription factor TFEB (Transcription Factor EB) (Wu et al., 2017). Without nuclear translocation of TFEB, the transcriptional activation of the MARCO promoter is blocked (Wu et al., 2017). IAV/bacterial coinfection also compromises AM viability (Palani et al., 2022). IFN-γ signaling in mononuclear phagocytes promotes the hyperproduction of TNF-α, associated with lethal lung inflammation at the later stage of coinfection (Verma et al., 2021; Palani et al., 2022).

The susceptibility to secondary infection can persist for months after the virus has been cleared, entering a state of sustained innate immune hypo-responsiveness (Didierlaurent et al., 2008). Resident AMs undergo long-term desensitization to bacterial Toll-like receptor (TLR) ligands (Didierlaurent et al., 2008). This state is primarily mediated by impaired NF-κB nuclear translocation upon secondary bacterial challenge, which prevents the rapid induction of pro-inflammatory cytokines and chemokines (Didierlaurent et al., 2008). During the resolving phase, immunosuppressive cytokines such as IL-10 and IL-27 are produced to curtail immunopathology, but they simultaneously deactivate bactericidal programs (Liu et al., 2014; Kelly et al., 2022). IL-10 inhibits the production of early protective IFN-γ by Type I NKT cells, while IL-27, acting downstream of Type I IFN, directly targets γδ T cells to decrease RORγt and IL-17 production (Cao et al., 2014; Barthelemy et al., 2017). This leaves a window of opportunity for unchecked bacterial outgrowth (Didierlaurent et al., 2008; Sencio et al., 2020).

### 2.3 Respiratory and intestinal microbiome dysbiosis

Influenza infection modulates the nasopharyngeal microbiome composition and initiates a rapid collapse of the respiratory microbial ecosystem by directly damaging epithelial cells and impairing mucociliary clearance (Pittet et al., 2010; Ding et al., 2019). This disruption follows the “Anna Karenina Principle,” which states that healthy microbiomes maintain a stable and uniform composition, whereas infected states exhibit highly unique, individualized, and dynamic dysbiosis patterns (Zaneveld et al., 2017; Kaul et al., 2020). During this destabilization, a “bloom” of opportunistic taxa, such as *Pseudomonadales*, is frequently observed (Kaul et al., 2020). Metagenomic analysis has confirmed that the actual growth rate of pathogenic bacteria is considerably accelerated during influenza infection, directly contributing to secondary bacterial complications (Li et al., 2023). Conversely, the pre-existing composition of the respiratory microbiome can influence host susceptibility to influenza infection (Lee et al., 2019). Specifically, an increased abundance of *Streptococcus* spp. and *Prevotella salivae* correlates with reduced susceptibility to influenza A (H3N2) virus, suggesting a protective role for certain commensal taxa (Tsang et al., 2020). Furthermore, the administration of *Clostridium butyricum* has been shown to alter the lung microbiome and enhance antiviral effects through IFN-*λ* upregulation in murine models (Hagihara et al., 2025).

Influenza affects not only the respiratory tract but also the intestinal ecosystem via the bidirectional “gut–lung axis” (Wang et al., 2014; Deriu et al., 2016; Yildiz et al., 2018). A pulmonary viral infection induces the systemic release of type I IFNs, which act as remote signals that alter the intestinal landscape (Deriu et al., 2016). Specifically, systemic Type I IFNs deplete beneficial intestinal anaerobes and promote the abnormal proliferation of *Proteobacteria*, thereby creating a dysbiotic microenvironment (Deriu et al., 2016). This shift inhibits local antimicrobial and inflammatory responses, enhancing intestinal colonization by *Salmonella* and its subsequent systemic dissemination (Deriu et al., 2016). This intestinal dysbiosis, compounded by infection-induced inappetence and reduced fiber intake, leads to a sharp decline in short-chain fatty acids (SCFA), particularly acetate (Sencio et al., 2020). Consequently, the host becomes highly susceptible to lethal secondary pneumococcal superinfection due to the loss of SCFA-mediated immune modulation (Sencio et al., 2020).

### 2.4 Roles of viral surface glycoproteins in promoting secondary bacterial infection

**Figure 3:**
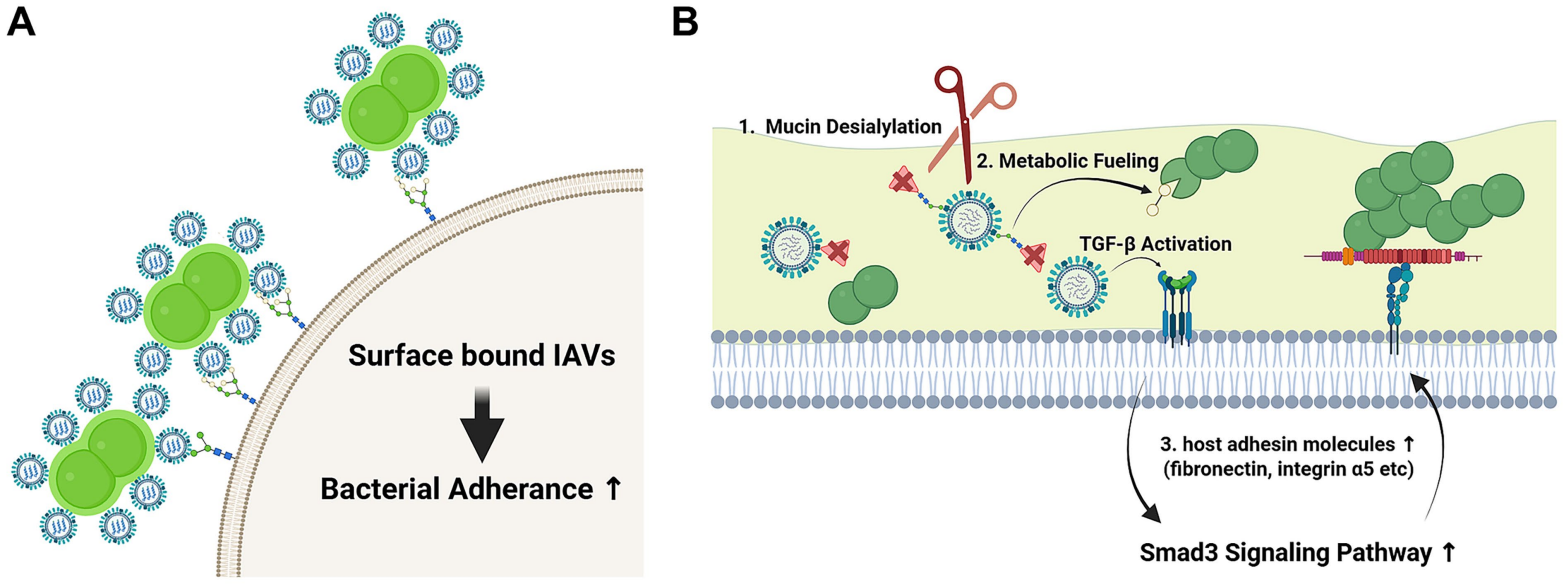
IAV surface glycoproteins HA and NA facilitate bacterial adherence and environmental remodeling. **(A)** HA-mediated physical tethering. Surface-bound IAVs, depicted as blue spiked particles, directly enhance bacterial adherence, represented by green spheres, by acting as a physical tether between the respiratory epithelial cell surface and bacterial pathogens. **(B)** NA-driven transformation of the respiratory environment. The viral NA glycoprotein promotes bacterial invasion through three interconnected pathways mediated by its enzymatic activity. (1) NA cleaves terminal sialic acid residues from respiratory mucins and cell-surface glycans to unmask cryptic receptors. (2) The liberated free sialic acid serves as a nutrient-rich carbon source that bacteria exploit for rapid proliferation, thereby creating a favorable metabolic niche within the nutrient-limited respiratory tract. (3) NA activates TGF-β to initiate the intracellular Smad3 signaling pathway, which leads to the transcriptional upregulation of host adhesin molecules such as fibronectin and integrin α5. These upregulated receptors provide stable anchoring sites that enhance the binding capacity of fibronectin-binding pathogens (created with Biorender.com).

The influenza virus and bacteria engage in complex physical and biochemical interactions that fundamentally change the course of respiratory disease (Siegel et al., 2014; Rowe et al., 2019). This synergy is primarily driven by the viral surface glycoproteins, hemagglutinin (HA) and NA, which act cooperatively to facilitate bacterial colonization and invasion (McCullers and Bartmess, 2003; Okamoto et al., 2003) (Figure 3).

Initially, HA promotes invasive disease by mediating direct physical tethering between the viral envelope and the bacterial surface (Okamoto et al., 2003; Rowe et al., 2019). While this interaction was traditionally highlighted in the context of the hyaluronic acid capsule of Group A *Streptococcus* (GAS), recent epidemiological and experimental observations have expanded this paradigm to a broader range of respiratory pathogens (Rowe et al., 2019). IAV has been shown to interact directly with both Gram-positive species, such as *S. pneumoniae* and *S. aureus*, and Gram-negative species, including *Moraxella catarrhalis* and non-typeable *H. influenzae* (Rowe et al., 2019). This direct binding facilitates a “Trojan horse” effect at the earliest stages of pathogenesis. Studies utilizing pre-incubation models have demonstrated that when IAV attaches to the bacterial surface, it significantly enhances bacterial adherence to respiratory epithelial cells and increases bacterial burdens *in vivo* (Okamoto et al., 2003). As the virus initiates its own entry via endocytosis, it inadvertently co-transports the attached bacteria into the intracellular environment, thereby allowing them to bypass primary host membrane barriers and establish severe infection (Okamoto et al., 2003). The significance of this physical axis is highlighted by studies showing that neutralizing HA with monoclonal antibodies effectively abrogates bacterial internalization and protects the host from lethal superinfection (Okamoto et al., 2003).

Parallel to these physical interactions, the NA glycoprotein the host microenvironment through its enzymatic activity (McCullers and Bartmess, 2003; Peltola et al., 2005). By cleaving terminal sialic acids from host glycoconjugates, NA transforms the respiratory landscape via three interconnected pathways (McCullers and Bartmess, 2003; Siegel et al., 2014). NA-mediated desialylation facilitates bacterial adherence by remodeling the structural architecture of the airway (McCullers and Bartmess, 2003; Peltola et al., 2005). In a healthy state, sialylated mucins serve as decoys that trap bacteria for clearance (Siegel et al., 2014). However, viral NA disrupts these decoys and strips sialic acid from the epithelial surface, thereby revealing underlying cryptic receptors such as laminin and collagen (McCullers and Bartmess, 2003; Peltola et al., 2005). This exposure provides pathogens such as *S. pneumoniae* with stable anchorage sites on the epithelium and the basement membrane (McCullers and Bartmess, 2003; Peltola et al., 2005; Duvigneau et al., 2016).

In addition to these structural alterations, the enzymatic byproducts of NA activity provide crucial metabolic support for bacterial outgrowth (Siegel et al., 2014). The liberation of free sialic acid from host surfaces creates a nutrient-rich niche within the otherwise nutrient-limited respiratory tract (Siegel et al., 2014). Pathogens including *S. pneumoniae*, *H. influenzae*, and *S. aureus* exploit this sugar as a preferential carbon source via specialized transporters, such as SatABC (Siegel et al., 2014). This metabolic advantage favors the rapid bacterial proliferation characteristic of post-viral pneumonia (Siegel et al., 2014). Concurrently, NA acts as a molecular trigger for pro-inflammatory signaling cascades (Li et al., 2015). By removing sialic acid motifs from the latent complex, NA activates transforming growth factor-β (TGF-β), which subsequently initiates the Smad3 signaling pathway (Li et al., 2015). This axis induces the transcriptional upregulation of host adhesins, specifically fibronectin and integrin α5, which enhances the binding capacity for fibronectin-binding bacteria such as *S. aureus* (Li et al., 2015; Bai et al., 2021). This signaling environment is further amplified by influenza-induced cyclophilin A (CypA), which activates FAK/Akt signaling to stimulate the actin rearrangement necessary for bacterial invasion (Bai et al., 2021). Collectively, these NA-driven mechanisms ensure that the host is not only structurally vulnerable but also metabolically and molecularly primed for severe secondary infection (Siegel et al., 2014; Bai et al., 2021).

### 2.5 Impairment of immunity and tissue repair by internal proteins

While surface glycoproteins initiate the infection process, viral internal proteins orchestrate a more profound dismantling of host defenses that sustains and exacerbates secondary bacterial infections (Conenello et al., 2011; Rowe et al., 2019). This post-viral landscape is potently regulated by non-structural protein 1 (NS1), which antagonizes innate immunity while disrupting cellular signaling required for tissue maintenance (Geiss et al., 2002; Hrincius et al., 2015). Primarily, NS1 acts as a potent antagonist of type I IFN signaling (García-Sastre et al., 1998). By interfering with the establishment of an “antiviral state,” it allows unchecked viral replication to prolong tissue injury (Geiss et al., 2002). In addition to immune evasion, the structural integrity of the lungs is directly undermined by NS1 through the inhibition of the signaling integrator c-Abl (Hrincius et al., 2015). This leads to severe lung pathology, massive edema, and the facilitation of secondary bacterial pneumonia (Hrincius et al., 2015). This structural insult is further compounded by a substantial “repair blockade” in which NS1 destroys progenitor basal epithelial cells and suppresses the expression of essential wound-healing factors, such as keratinocyte growth factor and hepatocyte growth factor (Kash et al., 2011). The resulting absence of proliferation markers, such as MCM7, effectively halts re-epithelialization (Kash et al., 2011). Consequently, the basement membrane and its cryptic receptors, including fibronectin and laminin, remain chronically exposed as stable platforms for bacterial anchorage (McCullers, 2006; Kash et al., 2011; Duvigneau et al., 2016).

PB1-F2 exacerbates pathology through mitochondrial targeting and inflammatory amplification (Zamarin et al., 2005; McAuley et al., 2007). It interacts with ANT3 and VDAC1 via its C-terminal mitochondrial targeting sequence (MTS) and induces BAK/BAX-mediated cytochrome c release, leading to the dissipation of mitochondrial membrane potential and subsequent pore formation (Zamarin et al., 2005; McAuley et al., 2010). This pro-apoptotic activity induces “immune paralysis” by specifically depleting alveolar macrophages and monocytes, thereby undermining the primary defense against bacterial invasion (Zamarin et al., 2005; Ghoneim et al., 2013). PB1-F2 also amplifies inflammation via the ERK1/2/AP-1 pathway, contributing to the induction of a cytokine storm. This pro-inflammation environment, fostered by PB1-F2, significantly promoted secondary bacterial infection. Notably, the full-length PB1-F2 of the 1918 strain induces severe purulonecrotic pneumonia, whereas the 2009 pandemic strain encodes a truncated, non-toxic version (McAuley et al., 2007; Sun et al., 2011). These distinct molecular characteristics provide a mechanistic basis for their differing pathogenic profiles and varying susceptibility to secondary bacterial infection (McAuley et al., 2007; Iverson et al., 2011).

### 2.6 Clinical association with MDR pathogens

Large-scale epidemiological studies indicate that influenza virus infections are significantly associated with an increased incidence of secondary infections caused by MDR bacteria (Lee et al., 2022; Gupta et al., 2022). For example, a study evaluating more than 8.2 million bacterial isolates from 257 US healthcare institutions revealed that community influenza rates were positively linked to the emergence of Gram-negative MDR strains (Gupta et al., 2022). Statistical models from this surveillance demonstrated that a 20% increase in the influenza rate was associated with an approximately 1% increase in fluoroquinolone-nonsusceptible *Enterobacterales* and *Pseudomonas aeruginosa* (Gupta et al., 2022). Furthermore, it was linked to a notable 4% increase in carbapenem-nonsusceptible *Acinetobacter* spp. (Gupta et al., 2022). Corroborating these trends, 16S rDNA sequencing of bronchoalveolar lavage fluid from adults with severe pneumonia identified *A. baumannii* as the most abundant bacterial species specifically within the influenza-positive cohort (Zhou et al., 2023).

The clinical implications of this correlation are particularly evident in cases of severe avian influenza A (H7N9) complicated by nosocomial *A. baumannii* coinfection (Liu et al., 2018). A meta-analysis of patients with H7N9 reported an overall *A. baumannii* coinfection incidence of 19.0%, which was associated with a fatality rate of 90.9% among cases with available outcome data (Liu et al., 2018). In a specific cohort of 24 patients with H7N9, 37.5% developed secondary *A. baumannii* pneumonia (Liu et al., 2018). This bacterial coinfection correlated with significantly prolonged reliance on invasive mechanical ventilation, persistent abnormalities in oxygenation indices, and consistently high chest radiograph scores post-onset. Longitudinal clinical data also demonstrated that these pathogens underwent rapid intra-host evolution, specifically evolving into pandrug-resistant strains within 5 days of targeted therapy (Liu et al., 2018).

These epidemiological trends and clinical observations provide objective evidence of a significant correlation between influenza virus infections and the emergence of severe MDR bacterial coinfections (Liu et al., 2018; Lee et al., 2022; Gupta et al., 2022). Although it is well-established that the influenza virus alters host immunity, the precise molecular and ecological dynamics driving the rapid selection of extreme drug resistance within the respiratory microbiome remain incompletely understood (Zhang et al., 2020; Li et al., 2023). Therefore, further research must systematically investigate the interaction mechanisms between influenza viruses and MDR pathogens to develop robust surveillance protocols and optimize therapeutic strategies for these complex coinfections (Zhang et al., 2020; Li et al., 2023; Zhou et al., 2023). The multifaceted interactions between influenza viruses and bacterial pathogens encompassing structural barriers, immune signaling, and the burgeoning threat of MDR species are summarized in Table 1.

**Table 1:** Summary of the multifaceted mechanisms underlying influenza-induced susceptibility to secondary bacterial infections.

Category	Mechanism		Pathogen	References
Remodeling of the respiratory surface	Structural disruption of the respiratory epithelium and Impairment of the mucociliary escalator		*S. pneumoniae*, *S. aureus*, *H. influenzae*, *P. aeruginosa*	Pittet et al. (2010), Iverson et al. (2011), McCullers (2014)
	Hijacking host cellular machinery		*S. pneumoniae*, *S. pyogenes*	Sumitomo et al. (2021)
Immune dysfunction	Inhibition of MARCO promoter transcriptional activation by IFN-γ		*S. pneumoniae*	Sun and Metzger (2008)
	Type I IFN-mediated disruption of the Th17/IL-17 axis and Inhibition of Cxcl1 expression leading to impaired neutrophil recruitment		*S. pneumoniae*, *S. aureus*, *E. coli*, *P. aeruginosa*	Shahangian et al. (2009), Kudva et al. (2011), Li et al. (2012), Lee et al. (2015)
	Innate effector functions of the remaining immune cells		*S. pneumoniae*, *S. aureus*	Small et al. (2010), Goulding et al. (2011), Ghoneim et al. (2013)
Microbiome dysbiosis	Bloom of opportunistic taxa according to the “Anna Karenina Principle”		*Pseudomonadales*, *Enterobacteriaceae*	Zaneveld et al. (2017), Kaul et al. (2020)
	Impact of the pre-existing composition of the respiratory microbiome		*S. pneumoniae*, *S. aureus*	Lee et al. (2019), Tsang et al. (2020)
	Intestinal systemic dissemination via the gut–lung axis		*S. pneumoniae*, *S. Typhimuriu* (Proteobacteria)	Deriu et al. (2016), Sencio et al. (2020)
Viral surface glycoproteins	HA-mediated direct physical tethering between the virus and specific bacterial species		*S. pneumoniae*, *S. aureus*, *S. pyogenes*, *Moraxella catarrhalis*, *Non-typeable H. inflluenzae*	Okamoto et al. (2003), Rowe et al. (2019)
	NA	Enhanced bacterial adherence via NA-mediated desialylation of host glycoconjugates	*S. pneumoniae*	McCullers and Bartmess (2003), Peltola et al. (2005)
		Metabolic support for bacterial outgrowth via enzymatic byproducts	*S. pneumoniae*, *S. aureus*, *H. influenzae*	Siegel et al. (2014)
		Activation of TGF-β-mediated transcriptional upregulation of host adhesins	*S. pneumoniae*, *S. aureus*, *S. pyogenes*, *H. influenzae*	Li et al. (2015)
		Induction of CypA-activated FAK/Akt signaling to drive actin rearrangement	*S. pyogenes*	Bai et al. (2021)
Viral internal proteins	NS1	Prolonged tissue injury via antagonism of type I IFN signaling	*S. pneumoniae*, *S. aureus*	Lee et al. (2010), Hrincius et al. (2015), Mikušová et al. (2022)
		Inhibition of c-Abl leading to acute lung injury and massive edema formation	*S. pneumoniae*	Hrincius et al. (2015)
		Halting of re-epithelialization leaving the basement membrane and its cryptic receptors chronically exposed	*S. pneumoniae*, *S. aureus*, *H. influenzae*	Kash et al. (2011), McCullers (2014)
	PB1-F2	Pro-apoptotic activity inducing “immune paralysis” through the depletion of AMs and monocytes	*S. pneumoniae*	McAuley et al. (2007), Ghoneim et al. (2013)
		Inflammatory amplification contributing to a “cytokine storm”	*S. pneumoniae*, *S. aureus*	McAuley et al. (2007), Zavitz et al. (2010), Iverson et al. (2011)
		Creation of an obstructed nutrient-rich niche for bacteria	*S. pneumoniae*, *S. aureus*, *S. pyogenes*	Pittet et al. (2010), McCullers (2014), Siegel et al. (2014)

## 3 Conclusion

The increased susceptibility to secondary bacterial infection following influenza results from a complex, synergistic cascade involving structural epithelial damage, profound immune dysregulation, and microbiome disruption. Understanding these multifaceted mechanisms has been fundamental to managing the clinical threat posed by primary community-acquired pathogens such as *S. pneumoniae* and *S. aureus*. However, these same physiological vulnerabilities clearly create a dangerous entry point for nosocomial pathogens, particularly *A. baumannii*. This shift in the microbial landscape represents a critical frontier in influenza research. It necessitates a decisive transition from traditional pathogen-centric approaches toward more holistic host–microbiome perspectives. Consequently, future therapeutic strategies must evolve to encompass interventions beyond the conventional administration of antivirals and antibiotics. Future research should prioritize the molecular mechanisms driving viral-bacterial synergism, with a particular focus on elucidating the specific correlation between influenza infection and the selective enrichment of MDR strains. Such studies will be vital for predicting and mitigating nosocomial outbreaks. Furthermore, uncovering the molecular basis of this selective enrichment is essential for managing MDR pathogens in the context of influenza. In conclusion, the medical community is tasked with addressing the lethal synergy between viral pathogenesis and bacterial resistance to develop robust defense mechanisms that will substantially enhance patient survival during both seasonal and pandemic influenza outbreaks.
